# Supplementary material for: Ambient Environmental Ozone and Variation of Fractional Exhaled Nitric Oxide (FeNO) in Hairdressers and Healthcare Workers
Source: Int J Environ Res Public Health. 2023 Feb 28;20(5):4271. doi: 10.3390/ijerph20054271 (PMC10001628; doi:10.3390/ijerph20054271)
Supplement: Supplementary file 1 [file ijerph-20-04271-s001.zip › ijerph-2170485-supplementary.pdf]

Ambient Environmental Ozone and Variation of Fractional Exhaled Nitric Oxide (FeNO) in Hairdressers and Healthcare Workers

Tonje Trulssen Hildre, Hilde Heiro, Ingvill Sandven and Bato Hammarström *

Environmental and Occupational Medicine, Department of Pulmonary Medicine, Division of Medicine, Oslo University Hospital., 0424 Oslo, Norway
* Correspondence: bato.hammarstrom@ous-hf.no

Supplement S1. Test for instrument repeatability. Measurements are FeNO values in ppb.

	Test person 1	Test person 2	Test person 3	Test person 4
Measurement 1	10	15	8	33
Measurement 2	11	14	10	24
Measurement 3	10	14	9	28
Measurement 4	11	13	11	24
Measurement 5	10	15	11	26
Mean	10.40	14.20	9.80	27.00
SD	0.49	0.75	1.17	3.35
CV	0.05	0.05	0.12	0.12

Supplement S2. Daily average air quality measurements including $PM_{2.5}$, PM_{10}, ozone, NO_2 and SO_2. *Negative SO_2 indicates very low or zero levels of pollutant.

September 17th-24th (Week 37-39)						
Date	$PM_{2.5}$	PM_{10}	Ozone	Ozone	NO_2	SO_2*
	μg/m³	μg/m³	μg/m³	ppb	μg/m³	μg/m³
Saturday Sep 17th	1.52	4.86	41.01	20.90	8.56	-0.15
Sunday Sep 18th	2.80	4.65	47.36	24.14	4.49	-0.70
Monday Sep 19th	1.73	4.26	52.38	26.69	4.87	-0.42
Tuesday Sep 20th	3.24	7.60	38.72	19.73	11.87	-0.71
Wednesday Sep 21st	5.13	11.95	20.30	10.35	20.94	-0.81
Thursday Sep 22nd	6.51	12.88	29.58	15.07	18.20	-0.42
Friday Sep 23rd	9.95	13.77	60.82	30.99	9.73	-0.40
Saturday Sep 24th	5.52	8.02	22.30	11.36	9.24	-0.16
Sunday Sep 25th	2.36	4.23	27.91	14.22	6.01	0.42
Monday Sep 26th	1.99	3.11	55.28	28.17	6.04	1.11
Tuesday Sep 27th	2.66	4.70	49.88	25.42	7.99	0.89
Wednesday Sep 28th	6.20	10.36	54.45	27.75	6.33	0.14
Thursday Sep 29th	5.39	9.06	36.14	18.42	9.51	-0.04

Supplement S3. Anonymized FeNO measurements (Day FeNO S/E) in ppb. After commuting and arriving at workplace (S). After ≥3 h work (E). Mean with correcting for symptoms (Mean-S). SD with correcting for symptoms (SD-S).

Week 38 Hairdressers FeNO S/ E												
	M S	M E	T S	T E	W S	W E	T S	T E	F S	F E	S S	SE
	8	9	8	6	10	8	7	9	7	8	11	14
	9	10	8	9	11	9	10	10	8	10	16	15
	12	11	13	13	13	13	10	11	9	10	16	17
	12	11	15	14	14	14	10	12	12	10	23	20
	15	13	18	17	14	14	11	12	14	15		
	20	18	19	19	16	15	12	16	14	15		
	23	20	22	21	17	19	15	16	15	17		
	23	20	22	22	21	19	15	17	16	21		
	25	22	22	22	21	21	16	18	18	21		
	26	24	24		21	21	16	23	20	22		
	27	27			24	23	23	23	22	24		
					25	24	23	26	28	24		
									41	42		
Mean	18.18	16.82	17.10	15.89	17.25	16.67	14.00	16.08	17.23	18.38	16.50	16.50
SD	7.14	6.27	5.88	5.80	5.05	5.28	5.06	5.60	9.24	9.09	4.93	2.65
Mean-S	18.57	17.00	17.00	15.00	19.09	16.10	14.20	15.44	15.44	15.91	16.67	16.33
SD-S	6.86	5.78	5.51	5.16	5.93	4.85	5.14	4.66	6.83	5.59	6.03	3.21

Week 39 Healthcare Workers FeNO S/ E										
	M S	M E	T S	T E	W S	W E	T S	T E	F S	F E
	7	7	9	6	9	5	8	9	8	6
	9	9	11	9	9	6	11	11	8	8
	9	9	12	12	11	10	12	14	8	12
	10	11	12	13	12	11	12	14	10	13
	10	12	12	13	13	13	13	15	11	13
	12	13	13	13	13	13	13	15	13	13
	12	13	13	14	15	15	14	15	13	14
	12	16	14	15	17	16	19	15	13	14
	14	16	14	15	18	17	20	18	14	16
	15	18	15	16	18	20	21	22	17	19
	16	20	17	18	22	22	25	26	17	23
	18	20	20	20	25	23	27	37	19	28
	18	22	31	23	26	24	45	41	25	30
	22	24	32	26	35	40			30	36
	34	41								
Mean	14.53	16.73	16.07	15.21	17.36	16.79	18.46	19.38	14.71	17.50
SD	6.76	8.41	7.05	5.25	7.44	8.92	9.82	9.76	6.50	8.69
Mean-S	14.46	16.77	16.67	15.50	16.33	15.42	16.25	17.92	14.27	16.73
SD-S	7.20	8.80	7.46	5.65	5.77	6.23	5.99	8.56	5.04	7.63

Supplement S4. Hairdressers FeNO measurements, sampling and commuting time. After commuting and arriving at workplace (FeNO S). After ≥3 h work (FeNO E). Distribution of data in histogram with normality tests.

Hairdressers					
		FeNO S (ppb)	FeNO E (ppb)	Minutes between	Commuting total min
N	Valid	62	61	61	62
	Missing	22	23	23	22
Mean		16.71	16.82	211.87	23.52
Median		16.00	17.00	194.00	25.00
Std. Deviation		6.53	6.29	39.70	10.67
Minimum		7	6	180	1
Maximum		41	42	337	55

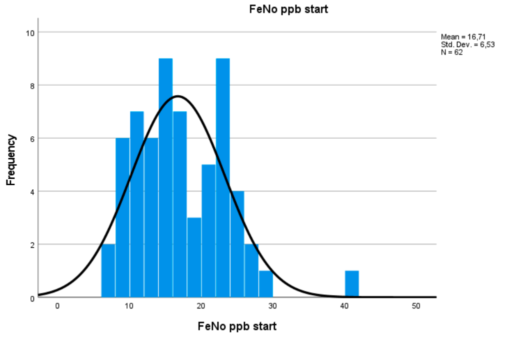

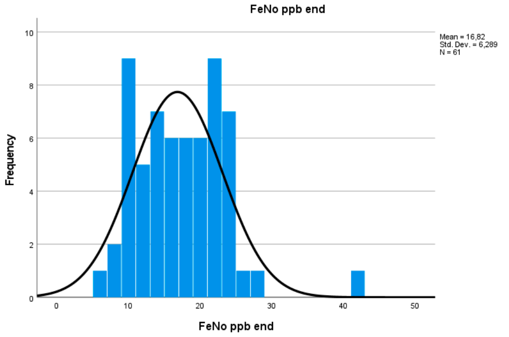

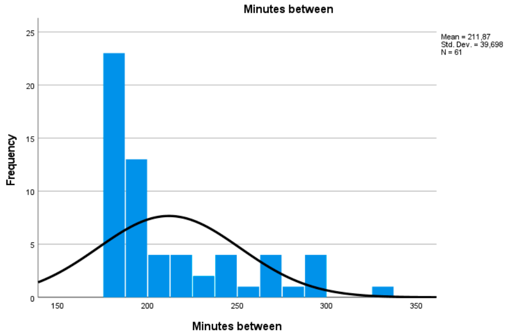

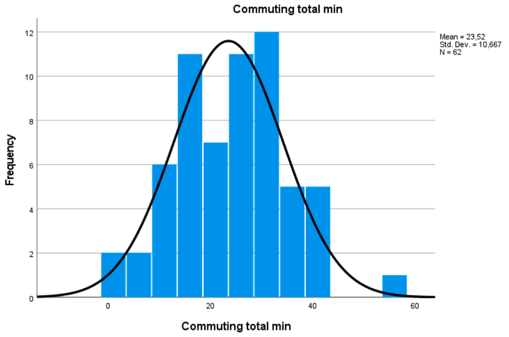

Tests of Normality						
	Kolmogorov-Smirnov[a]			Shapiro-Wilk		
	Statistic	df	Sig.	Statistic	df	Sig.
FeNO S	.120	61	.028	.939	61	.004
FeNO E	.078	61	.200*	.929	61	.002
Minutes between	.227	61	<.001	.793	61	<.001
Commuting total min	.105	61	.095	.974	61	.215
*. This is a lower bound of the true significance.						
a. Lilliefors Significance Correction						

Supplement S5. Healthcare workers FeNO measurements, sampling and commuting time. After commuting and arriving at workplace (FeNO S). After ≥3 h work (FeNO E). Distribution of data in histogram with normality tests.

Healthcare workers					
		FeNO S (ppb)	FeNO E (ppb)	Minutes between samples	Commuting total min
N	Valid	70	70	70	70
	Missing	5	5	5	5
Mean		16.17	17.09	220.04	41.03
Median		13.50	15.00	194.50	30.00
Std. Deviation		7.49	8.19	41.66	28.02
Minimum		7	5	180	4
Maximum		45	41	317	120

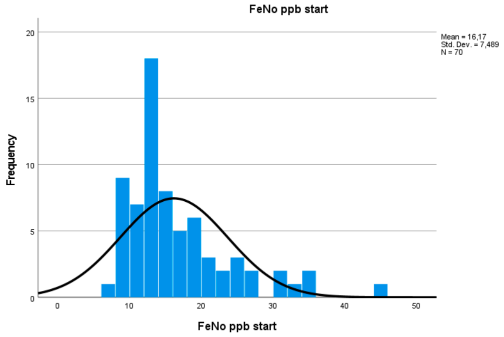

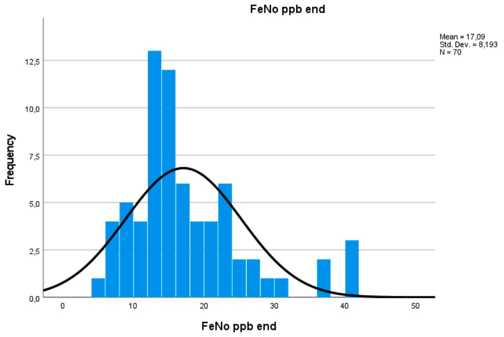

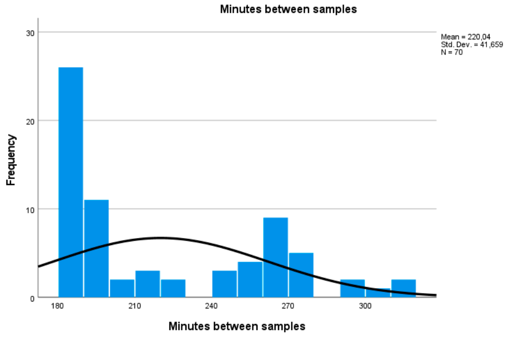

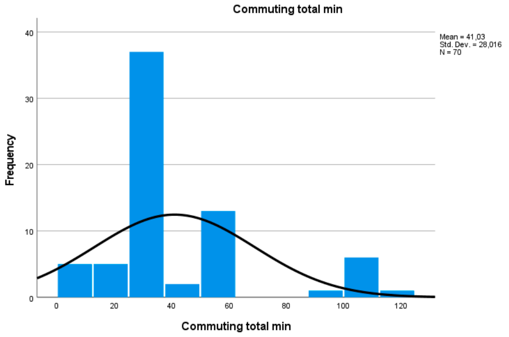

Tests of Normality						
	Kolmogorov-Smirnov[a]			Shapiro-Wilk		
	Statistic	df	Sig.	Statistic	df	Sig.
FeNO S	.185	70	<.001	.854	70	<.001
FeNO E	.181	70	<.001	.882	70	<.001
Minutes between samples	.247	70	<.001	.833	70	<.001
Commuting total min	.310	70	<.001	.809	70	<.001
a. Lilliefors Significance Correction						

Supplement S6. Hairdressers and healthcare workers symptoms, commuting and treatments. After commuting and arriving at workplace (FeNO S). After ≥3 h work (FeNO E). (n) represents the daily number of measurements in the FeNO S group. N=No and Y=Yes. *P-values are calculated with Student's t-test, otherwise with Mann-Whitney test. ^Time in minutes.

	n	FeNO S	FeNO E	p-value	Sampling^	Commuting^	p-value S	p-value E
Symptoms								
Short of breath Y	0							
Fever Y	0							
Cough Y	10	22.50	23.10	0.984	221.8	27.1	0.303	0.431
Cough N	122	15.95	16.45	0.446	215.8	33.3		
Cold Y	29	19.83	20.86	0.090*	203.9	22.0	0.018	0.004
Cold N	103	15.49	15.95	0.536	219.7	36.0		
Commuting								
Pedestrian Y	64	17.36	17.91	0.131*	216.6	15.2	0.026	0.040
Pedestrian N	68	15.54	16.06	0.469	215.8			
Bicycling Y	13	15.23	14.00	0.190*	216.4	41.5	0.601	0.110
Bicycling N	119	16.55	17.29	0.331	216.2			
Public transport Y	76	16.55	16.91	0.780	220.1	23.0	0.743	0.855
Public transport N	56	16.25	17.04	0.373	211.1			
Car Y	35	17.54	18.03	0.754	218.2	18.8	0.319	0.310
Car N	97	16.02	16.57	0.592	215.5			
Smoking/vaping Y	6	16.17	16.50	0.375*	188.7	21.3	0.858	0.468
Smoking/vaping N	126	16.44	16.98	0.468	217.6	33.4		
Treatments								
Bleach Y	29	14.55	14.83	0.292*	218.7	24.1	0.007*	0.009*
Bleach N	33	18.78	18.63	0.415*	205.7	22.9		
Dyes Y	30	14.83	14.83	0.500*	220.5	24.0	0.014*	0.007*
Dyes N	32	18.65	18.74	0.463*	203.5	23.9		
Permanent Y	1	15.00	13.00		294.0	25.0		
Permanent N	61	16.74	16.88		210.5	23.5		
Spray Y	35	17.63	17.46	0.349*	219.5	21.3	0.105*	0.181*
Spray N	27	15.62	15.96	0.311*	201.6	26.3		
Other Y	12	18.92	16.83	0.054*	227.1	27.6	0.097*	0.497*
Other N	50	16.24	16.82	0.119*	210.0	22.6		
